# Factors associated with Diabetes Empowerment among patients with type 2 diabetes, at OPD setting, Karachi, Pakistan

**DOI:** 10.1038/s41598-023-34385-4

**Published:** 2023-05-03

**Authors:** Mohiba Ali Khowaja, Shafquat Rozi, Sobiya Sawani, Asma Ahmed

**Affiliations:** 1 Department of Community Health Sciences, Aga Khan University, Karachi, Pakistan; 2 Department of Endocrinology, Aga Khan University Hospital, Karachi, Pakistan; 3 Pakistan Society of Pediatric Oncology, Karachi, Pakistan

**Keywords:** Diseases, Endocrinology, Health care, Medical research, Risk factors

## Abstract

Diabetes Empowerment is important for diabetic control as it postpones the onset of complications. This study aimed to investigate the association of medication adherence, self-care behaviors, and diabetes knowledge with Diabetes Empowerment among patients with type II diabetes. A cross-sectional study was conducted on 451 type II diabetes patients attending Endocrinology clinics at OPD setting in Karachi. Data was collected electronically using a structured questionnaire comprising of tools to measure Diabetes Empowerment, medication adherence, self-care behaviors, diabetes knowledge, and socioeconomic scale. It also included health-related information from patients’ medical record. As outcome variable was continuous, so multiple linear regression analysis was used to assess the independent effect of Diabetes Empowerment on medication adherence, self-care behaviors and diabetes knowledge with other co-variates. The mean Diabetes Empowerment score was 3.62 (SD = 0.31). The mean age of the participants was 56.68 (SD = 11.76). 53.88% were females, 80.71% were married, 77.56% were obese, and 66.30% were upper-middle class with average diabetes duration of 11.7 years (SD = 7.89). HbA1c values were ≥ 7 in 63.41% of study participants. Diabetes Empowerment was significantly associated with medication adherence (P = 0.001), general diet (P < 0.001), special diet (P = 0.011), smoking status (P = 0.001), and socioeconomic status (upper lower, P = 0.085). A comprehensive strategy for the treatment of type II diabetes is essential to enhance clinical results, improve patient quality of life, and prevent diabetes-related comorbidities. People with type II diabetes should be encouraged to embrace an empowerment-based approach by healthcare providers. It is critical to do research that promotes empowerment.

## Introduction

Diabetes Mellitus is a chronic metabolic disorder that occurs when either pancreas does not produce insulin or when resistance to insulin is developed in the body^1^. Uncontrolled diabetes causes long term damage to the eyes, kidneys, nerves, heart, and blood vessels^2^. In 1980, 108 million people were diagnosed with diabetes, which has increased to 422 million adults in 2014, according to the World Health Organization (WHO)^3^. Diabetes affects 19.4 million people in Pakistan, and it is expected to reach 37.1 million by 2045^4^.

Poor medication adherence, inadequate diabetes knowledge, and poor self-care behaviors can lead to high blood glucose levels^5^. Diabetes can be managed by lifestyle changes, medication adherence, and knowledge about the disease^4^.

First, medication adherence is a person's willingness to take their prescribed medications^6^. Non-adherence to medications is often caused by forgetfulness, drugs interfering with meals, lack of financial support, drug ineffectiveness, side effects, multiple medications, being too busy to take them, and symptoms disappearing^7, 8^. A previous study in Pakistan found that 72% of participants had low medication adherence^5^. Second, self-care behaviors includes diet, exercise, foot care, blood glucose monitoring, and smoking. A Pakistani study found that 9.5% of participants did not monitor glucose levels, 25% did not fully follow dietary advice, 43.9% did not practice foot care, and 61.7% did not exercise despite proper understanding about it^9^. Finally, diabetes knowledge helps patients understand diabetes and the consequences of not receiving proper treatment^10^. According to a 2015 Pakistani study of 392 patients with diabetes, 62.5% had average disease knowledge^5^. Previous studies have shown that diabetic knowledge increases medication adherence and self-care behaviors, which helps manage diabetes^11, 12^.

A comprehensive approach for type II diabetes management is required, which includes Diabetes Education, an emphasis on lifestyle changes, achieving good glycemic control, avoiding drugs that can aggravate glucose or lipid metabolism, and screening for diabetes complications^13^. For the management of chronic illnesses like as diabetes, it is important to perform empowerment-based research.

Empowerment is taking responsibility for one's health and lifestyle^14^. Diabetes Empowerment enables patients to make informed decisions and discover their capability to manage one’s diabetes^14^. In multiple studies it has been seen that Diabetes Empowerment has a very positive effect in reduction of HbA1c and maintaining self-care behaviors, hence controlling overall health of the individual with diabetes^15, 16^. In a health-care system, that of Pakistan the treatment measures totally depend on the physicians and a little interest is taken by the patients regarding their own health^5^. In this setup, patients should be empowered to manage their diabetes since this strategy can help them avoid complications and enhance their overall quality of life^13^.

According to previous studies empowerment is influenced by cultural and social norms^17^, so it is important to look for factors that influence diabetes knowledge within different societies. This study will access the acceptability of empowerment among patients with type II diabetes regarding their own health in Pakistan. To the best of our knowledge, no such study has been conducted in Pakistan, to look for factors like diabetes knowledge, medication adherence and self-care behaviors, and their association with Diabetes Empowerment, so this study will provide us the base line data regarding Diabetes Empowerment. This will be helpful in identifying the underlying gaps, designing interventional studies for improvement in patient’s health and can help in developing new policies and make future action plans for diabetes control.

This study aims to evaluate the relation of predictor variables including medication adherence, self-care behaviors and diabetes knowledge with Diabetes Empowerment among patients with type II diabetes mellitus visiting Endocrinology clinics at OPD setting, in Karachi Pakistan.

## Material and methods

### Study design and study setting

A cross sectional study design was employed, as our study objective was to check the association of medication adherence, self-care behaviors, and diabetes knowledge with Diabetes Empowerment in patients with diabetes of Pakistan.

The study was conducted in outpatient clinics of Endocrinology at Aga Khan University which is a private, not-for-profit system with some subsidiary or welfare support hospital in Karachi Pakistan. The reason for the selection of outpatient Endocrinology clinic of AKU is that the turnover of AKU Endocrinology clinic is 305 patients per day out of which 80% are patients with type II diabetes mellitus, along with these people from all over Pakistan with different ethnicities come to AKU for their treatment.

### Study population, sampling technique, and sample size

All participants coming to Endocrinology clinics for their regular appointments from September 23rd to October 23rd 2021 were enrolled in the study. All included participants were above 18 years, diagnosed cases of type 2 diabetes for more than a year, taking oral hypoglycemic agents with or without insulin per day. Patients were excluded if they did not understand English or Urdu language.

Participants were recruited through non-probability purposive sampling technique. A minimum sample of 451 patients with diabetes were needed to achieve the study objectives, keeping 5% level of significance, 80% power and adjusting for 10% non-respondent. In clinic waiting rooms, all eligible patients were approached.

### Variables and tools

Data was collected through a structured questionnaire, comprising sociodemographic variables and health-related variables. Validated questionnaires were used to assess all the main exposure variables i.e. medication adherence, self-care behaviors, and diabetes knowledge.

### Demographic and health-related variables

Sociodemographic variables include age, gender, religion, ethnicity and marital status. Socioeconomic status was assessed using the Modified Kuppus Wammy scale, which is a valid questionnaire with three parameters including occupation, education, and income^18^.

Health related variables include duration of diabetes, HbA1c levels^19^ and body mass index of patients. The body mass index (BMI) was calculated using heights and weights of the patients^20^.

### Medication adherence

Medication adherence is one of the main exposure variables. It is a categorical variable and is assessed through a valid questionnaire Brief Medication Questionnaire^21^. Brief Medication questionnaire is based on four screens, “Regimen screen, Belief screen, Recall screen and Access screen”^21^. In this study we used all four screens to oversee explicit kinds of non-adherence or hindrances to adherence to medications^21^. The sensitivity and specificity of regimen screen is 80% and 100%, for belief screen is 80% and 62% and for recall screen is 90% and 80% respectively for all screens^21^. A positive screen indicates that the patient reported some medication non-adherence or hurdles to adherence, whereas a negative screen indicates that the patient reported complete medication adherence or no barriers to adherence^21^.

### Self-care behaviors

Self-care behaviors was the second main exposure variable. Diet, exercise, monitoring of blood sugar levels, and foot care are some important factors of self-care behaviors^22^. Self-care activities were calculated with a valid questionnaire by The Summary of Diabetes Self-care activities measures^23, 24^. There are eleven items in this questionnaire. The variables of this scale are quantitative. Responses were calculated on basis of the last seven days of the week. Later mean of questions were taken to evaluate general diet (Q1 and 2), special diet (Q3 and 4), exercise (Q5 and 6), blood glucose monitoring (Q7 and 8), foot care (Q9 and 10), and smoking (Q11) separately^23^.

### Diabetes knowledge

In this study, knowledge was defined as the ability to comprehend diabetes-related information^10^. Diabetes knowledge is another main exposure variable. Diabetes knowledge was calculated with questionnaire by The Starr County, which is a valid questionnaire^25, 26^. This questionnaire has 24 questions. For each correct answer the person receives a score of 1 and of all incorrect answer the person receives a zero score^25^. In these 24 questions, if a person scores > 12 so it is considered that the person has adequate or good knowledge about Diabetes and if a person scores < 12 are considered as inadequate or bad knowledge about diabetes^25^. This variable is a qualitative variables with categories of adequate knowledge and inadequate knowledge^25^.

### Diabetes Empowerment

Diabetes Empowerment is the outcome variable. Diabetes Empowerment is defined as the ability to make decisions about one's illness management with the support of adequate knowledge and resources^27^. In this study, Diabetes Empowerment was measured with a validated tool by Michigan Research Center, Diabetes Empowerment Scale—Short form (DES-SF) which is an eight-item questionnaire^28^. The reliability of the Diabetes Empowerment scale—short form showed α = 0.85 while using the original data set^28^. The score of the scale was obtained by adding up all the answers and then dividing it by the total number of the questionnaire^29^. This variable is a quantitative variable with a minimum score of 1 and the maximum score of 5^29^.

As the outcome, the Diabetes Empowerment scale is validated and used regionally tool in China^30^ but has not been used in Pakistan, so we required content validity of the scale. A panel of experts with a background in endocrinology and research assessed the tool of Diabetes Empowerment before this study began.

### Data collection procedure

Hiring of data collector was done for a time of 1 month. The training of the data collector included teaching the data collector procedure of taking informed consent, filling of google-form based structured questionnaire using smart phone. A manual of operation was provided to the data collector to facilitate consistency in protocol implementation for recruiting participants of study, implementing the desired tools, collecting data and analyzing the data. Health related information was collected by investigator using medical record files. As this study was in form of a questionnaire, we provided each participant with pamphlets regarding information of Diabetes Empowerment. The pamphlet was made for all those participants who completed the questionnaire. It was sent separately through WhatsApp application in PDF format in both English and Urdu languages.

### Ethical clearance

An Ethical review was sought from the Departmental Review Board (DRC). After receiving DRC approval, The IRB: Ethics and Review Committee of Aga Khan University, Karachi, was consulted for ethical permission. The ethical review board of The Aga Khan University Hospital approved the study (reference number: 2021-6556-19261). The data collector informed the patients who met the criteria of inclusion and gave their consent, then the structured questionnaire was filled using information from the participants.

### Statistical analysis

Diabetes Empowerment, which is the outcome variable is a continuous variable and calculated on basis of scores. Multiple linear regression analysis was performed using STATA version 16 software.

The descriptive statistics of all independent variables and outcome variable was conducted. Mean and standard deviation of all normally distributed continuous variables like age, self-care activities were reported. Median and Interquartile ranges (IQR) of all skewed continuous variables were reported. Frequency and percentages of all categorical variables like gender, ethnicity, education level, employment status, SES, and diabetes knowledge were reported. The univariate analysis was performed using simple linear regression analysis. Each variable was regressed separately with Diabetes Empowerment. Linear regression coefficients, standard errors and 95% confidence intervals were reported. The significance was assessed at a p-value of ≤ 0.25. Such variable with p-values greater, were removed and other variables with significant p-values were included in multivariable analysis. In Multivariable analysis, all variables that were significant at univariate analysis level, these were assessed using multiple linear regression analysis. All variables with a p-value ≤ 0.05 were added in the model. The presence of biologically plausible interaction and confounding was also assessed. The final model was evaluated for adequacy by normal probability plots, Shapiro–Wilk Test and residuals versus fitted plots. The outliers and influential were checked by Standardized residuals, leverages, cook’s distance, DFBETA and DFIT.

### Data management

The data was collected by a web-based questionnaire on google forms. The data was assessed for accuracy and completeness on daily basis by investigator. Range checks and internal consistency checks were already built-in google forms. In the google forms, all the questions were marked mandatory, so the participants were not able to move further without answering the questions. Hence, the issue of missing or incomplete data was not there.

In order to ensure that the data was not misplaced, backup files were created. To ensure confidentiality, backup files didn’t have participant’s name and contact details. Data was stored in such a manner that each participant was provided with unique ID numbers. The Electronic data has been secured with a password and this data will be discarded after seven years of completion of the study as per university policy. Strict measures were taken to minimize the data handling and movement.

### Ethics approval and consent to participate

Ethical approval was taken from The Aga Khan University Ethical Review Committee (2021-6556-19261). All procedures performed in studies involving human participants were in accordance with the ethical standards of the institutional committee.

### Informed consent

Before data collection (access to electronic form), all participants were asked to sign a form of consent to be included in this study.

## Results

**Figure 1:**
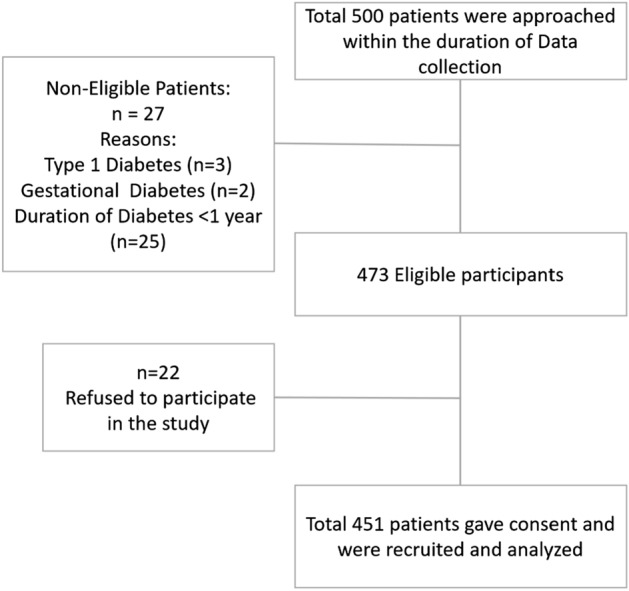
Flow chart of the participants.

During the month of data collection, 500 patients attended AKUH for regular Endocrinology Clinic visits and filled the screening questionnaire. After screening for eligibility criteria, 451 individuals with type II diabetes were recruited. Around 22 individuals declined to participate in the study, while 27 others were ruled out because they were not eligible (Fig. 1).

Description of sociodemographic characteristics, health related information, medication adherence, self-care behaviors and diabetes knowledge of the study population is shown in Tables 1 and 2. The study enrolled 451 patients with type II diabetes mellitus to test Diabetes Empowerment. Medication adherence, self-care behaviors (general diet, special diet, exercise, blood glucose monitoring, foot care, and smoking status), and diabetes knowledge are the main exposure variables. Other covariates include sociodemographic characteristics (age, ethnicity, religion, marital status, and socioeconomic status) and health-related information (diabetes duration, HbA1c levels, and BMI) (Table 1).

**Table 1:** Sociodemographic characteristics and health related information of n = 451 patients with diabetes recruited in the study.

Variables	n (%)
Sociodemographic factors	
Age (Mean ± SD)	56.68 ± 11.76
Gender	
Female	243 (53.88)
Male	208 (46.12)
Ethnicity	
Urdu	193 (42.79)
Sindhi	119 (26.39)
Punjabi	46 (10.20)
Others	93 (20.62)
Religion	
Islam	438 (97.12)
Others	13 (2.88)
Marital status	
Single	17 (3.77)
Married	364 (80.71)
Divorced/widowed	70 (15.52)
Socioeconomic status	
Upper lower	9 (2)
Lower middle	46 (10.20)
Upper middle	299 (66.30)
Upper	97 (21.51)
Health related information	
Duration of diabetes (Mean ± SD)	11.7 ± 7.98
HbA1c levels	
<= 7 mmol/mol	165 (36.59)
> 7 mmol/mol	286 (63.41)
Body mass index	
Underweight	3 (0.67)
Normal Weight	44 (9.78)
Overweight	54 (12)
Obese	349 (77.56)

HbA1c, glycosylated hemoglobin.

**Table 2:** Diabetes Empowerment, medication adherence, self-care behaviors and diabetes knowledge of n = 451 patients with diabetes recruited in the study.

Variables	n (%)
Diabetes empowerment (Mean ± SD)	3.62 ± 0.31
Primary exposures	
Medication adherence	
Adherence	371 (82.26)
Non adherence	80 (17.74)
Self-care activities	
General diet (Mean ± SD)	6.26 ± 1.52
Special diet (Mean ± SD)	4.16 ± 0.93
Exercise (Mean ± SD)	1.66 ± 1.84
Blood glucose monitoring (Mean ± SD)	4.81 ± 2.28
Foot care (Mean ± SD)	6.49 ± 1.71
Smoking	
Non Smoker	428 (94.90)
Smoker	23 (5.10)
Diabetes knowledge	
Inadequate knowledge	15 (3.33)
Adequate knowledge	436 (96.67)

The mean age of patients with type II diabetes mellitus was 56.68 (SD = 11.76) years. There were more female participants 243 (53.88%), married 364 (80.71%), Muslims 438 (97.12%), Urdu speaking 193 (42.79%). The mean duration of Diabetes 11.7(SD = 7.98) years among participants. Majority of participants belong to Upper middle socioeconomic group 299 (66.30%). Most of the patients with diabetes were obese 349 (77.56%) with Hemoglobin A1c greater than 7 i.e. 286 mmol/mol (63.41%). The mean height of all patients was 160.88 cm (SD = 9.07) and mean weight is 76.17 kg (SD = 16.52) (Table 1).

The mean Diabetes Empowerment measured among participants was 3.62 ± 0.31. Around 82% participants were adhered to medications and had no barriers. The mean scores of self-care behaviors was for general diet 6.26 (SD = 1.52), for special diet 4.16 (SD = 0.93), for exercise 1.66 (SD = 1.84), for Blood Glucose Monitoring 4.81 (SD = 2.28) and for foot care 6.49 (SD = 1.71) maintained in the last 7 days. The average population that smoked was 5.10%. Around 436 (96.67%) individuals had adequate knowledge about Diabetes (Table 2).

Multiple linear regression (MLR) was used to identify variables that contribute to outcome (Diabetes Empowerment), with a p-value of 0.05 considered statistically significant in a step-wise manner. General diet, special diet, Medication Adherence, Smoking Status, foot care, socioeconomic status and exercise were associated with overall Diabetes Empowerment score (Table 3). All other primary exposure variables were also kept in the model.

**Table 3:** Multivariable analysis of factors associated with overall Diabetes Empowerment Scale of n = 451 patients with diabetes recruited in the study.

Variable	β coefficient	Confidence interval
General diet	0.043	0.021 to 0.066
Special diet	0.039	0.009 to 0.069
Medication adherence		
Adherence (Ref)	–	–
Non-adherence	− 0.125	− 0.207 to − 0.042
Smoking status		
Non-smoker	–	–
Smoker	− 0.638	− 0.998 to − 0.278
Blood glucose testing	0.006	− 0.006 to 0.018
Foot care	− 0.014	− 0.039 to 0.011
Socioecomic status		
Upper lower	0.183	− 0.026 to 0.391
Lower middle	0.03	− 0.112 to 0.106
Upper middle	0.028	− 0.039 to 0.095
Upper (Ref)	–	–
Exercise	− 0.007	− 0.022 to − 0.008
Diabetes knowledge		
Adequate knowledge (Ref)	–	–
Inadequate knowledge	0.108	− 0.085 to 0.301
Smoker * foot care	0.088	0.033 to 0.144

One statistical interaction between the smoking status and foot care was found to be significant and was kept in the model. Model was then assessed for confounding factors but none of the variables showed positive confounding.

The overall Diabetes Empowerment scores increases for every one day increase in practice to general diet by 0.043 (95% CI: 0.021–0.065). For every one day increase in special diet practice, the overall Diabetes Empowerment score increases by 0.039 (95% CI: 0.009–0.069), keeping all other variables constant. Diabetes Empowerment decreases with increase in non-adherence to medications by 0.125 (95% CI: − 0.208 to − 0.042). Among smokers the mean estimated difference of overall Diabetes Empowerment decreases by 0.638 (95% CI: − 0.998 to − 0.278) as compared to non-smokers when adjusting for all other variables. Among patients from upper lower class the mean estimated difference of the overall Diabetes Empowerment score increases by 0.183 (95% CI: − 0.026 to 0.391) (Table 3).

The difference in estimated mean Diabetes Empowerment scores is 0.088 between smokers and non-smokers, when days of foot care increases by one day, adjusting for all other variables (Table 3).

## Discussion

This is the first detailed study conducted at an Endocrinology Clinic in Pakistan to identify the association of Medication Adherence, Self-care Behavior and Diabetes Knowledge with Diabetes Empowerment among 451 patients in Karachi. Medication Adherence, general diet, special diet, foot care, Diabetes Knowledge, age, marital status and socioeconomic status are strong predictors of Diabetes Empowerment. The mean overall score of Diabetes Empowerment among Pakistani population was 3.62 (SD = 0.31). The results of the study are favorable and almost similar as compared to other regional studies that have used the same tool for Diabetes Empowerment^30–32^.

Diabetes treatment modalities include lifestyle modifications, exercise, medication adherence and diabetes knowledge^33^. According to our study findings, diabetes empowerment was associated with general diet, medication adherence, special diet, foot care, smoking, and diabetes knowledge.

Patients' performance in all facets of self-care behaviors has been variable. In our study, with increase in maintaining general diet in past 7 days there is a significant increase in empowerment of a patient suffering from type II Diabetes. The findings are congruent with those of previous investigations both on international^27^ and regional level studies^30, 32^. In our study, following a specific diet for the last seven days resulted in a considerable boost in the empowerment of a type II diabetes patient. Our results and those of Hernandez-Tejada^27^, suggesting that by maintaining good special diet in past seven days, empowerment among patients increases.

With increased adherence, patient’s empowerment also increases. As seen in the results of our study, increased non-adherence decreases diabetes empowerment among patients with type II diabetes mellitus of Pakistan. The results of our study are consistent with previous studies, showing that with increased medication adherence empowerment among patients with type II diabetes mellitus increases^27^.

Our study findings showed that with increase in knowledge regarding diabetes, diabetes empowerment also increases. Among patients with diabetes, knowledge about the disease increases one’s empowerment regarding disease. The results of our study are consistent with other studies^17, 27, 30, 34^.

Our study shows interaction between Foot care and Smoking status as a cause of diabetes neuropathy that among those who smoke have difference in mean estimated overall Diabetes Empowerment scores 0.088 times more as compared to non-smokers, when days of foot care increases by one. Patients who are smokers may lead to peripheral diabetes neuropathy in patients with Diabetes causing decrease in empowerment scores. Findings of our study are consistent with other studies, in which patients with smoking habit and decreased foot care habits can be a cause of peripheral diabetes neuropathy^35^.

Our Study also found that among upper lower class, lower middle class and upper middle class, diabetes empowerment increases as compared to Upper class individuals. According to previous studies individuals from low socioeconomic status have poorer diabetic control as compared to higher socioeconomic status, which are inconsistent with our results^36^. Patients with low socioeconomic status are found to be less adhered to medications, life style modifications and knowledge which leads to poor glycemic control^36^.

Among patients coming to AKUH, Diabetes empowerment decreases with increase in exercise routine in last seven days. These results are inconsistent with the previous findings as other studies and literature both suggest that with increase of exercise in daily routine, diabetes empowerment increases^27, 30, 34^. However, in our study limited patients follow recommended exercise routine in our population, it may be a cause of such relationship. We also observed inconsistent relationship of blood glucose monitoring and Diabetes Empowerment. According to our findings, blood glucose monitoring is inversely related with Diabetes Empowerment. These findings are not in congruent with literature^27, 30, 34^. Hence, it is possible that the patients of our study were not following proper blood glucose monitoring regimen as per recommendation of the Physicians.

### Strengths and limitations

Patient empowerment was not emphasized in traditional diabetes teaching^30^. This study has shown to be beneficial since, to our knowledge, no prior study in Pakistan has identified relationship of medication adherence, self-care behaviors and diabetes knowledge with diabetes empowerment among patients with type II diabetes mellitus. Secondly, data collector was trained to fill out the questionnaire accurately under the investigator's supervision. Strict ethical criteria were followed throughout the questionnaire filling procedure. Thirdly, the questionnaire was developed using valid, reliable, and standardized tools. Finally, AKUH Endocrinology Clinics treated patients from all ethnicities, socioeconomic classes, and Pakistani provinces during the research period.

There are certain limitations to this study that are worth mentioning. First, this was a cross-sectional study; therefore, it was unable to determine whether medication adherence, self-care behaviors, and diabetes education were causally associated with diabetes empowerment in longitudinal fashion. We only looked into the relationship of all predictor variables with diabetic empowerment. Second, severity of disease, self-efficacy, comorbidities, cultural and societal norms, and medication adherence, self-care, and diabetes education may affect diabetes empowerment. Third, employing scales to collect data on diabetic self-care and medication adherence may have caused recall bias, limiting the study's findings. Fourthly, the study was limited to AKUH, therefore we cannot generalize the results. Finally, we eliminated those with psychiatric issues including depression, anxiety, and stress, which might hinder patient empowerment. Future researches should address these shortcomings.

### Implications for practice

A comprehensive approach for type II diabetes management is required, which includes Diabetic education, lifestyle adjustments, glycemic control, cardiovascular disease risk reduction, medicine avoidance, and screening for diabetes complications are needed^13^. First, doctors should empower patients with type II diabetes mellitus and their caregivers. This may reduce diabetic nephropathy, neuropathy, and retinopathy. Empowering diabetics can minimize complications and enhance quality of life^13^. Diabetes empowerment education should be culturally and realistically tailored. Social media and diabetic empowerment videos can be utilized for this.

As this is a single hospital-based cross-sectional study, researchers should conduct additional longitudinal studies at several hospitals and clinics to identify and educate people about diabetes empowerment. Further to understand diabetes empowerment's predictive characteristics, qualitative research is required. Diabetes empowerment can also be influenced by illness severity, comorbidities, social and cultural norms, and self-efficacy. Diabetes empowerment is affected by several factors. Instead of taking an authoritative approach to chronic illnesses like diabetes, health care practitioners should focus on empowering people. Future randomized control trials should investigate the impact of diabetic empowerment interventions on health habits, complications, and quality of life.

## Conclusion

Comprehensive diabetes care is essential. This strategy requires patients to take charge of their diabetes care. Diabetes empowerment is a beneficial and therapeutically relevant method for treating diabetes, improving glycemic control, and reducing complications. Medication adherence, self-care behaviors such general diet, special diet, exercise, blood glucose monitoring, foot care, and smoking status, as well as Diabetes education, predict Diabetes Empowerment and help manage diabetes patients in Pakistan. In future randomized controlled trials should be conducted to compare whether intervention helps to improve the quality of life of patient with Diabetes and control HbA1c levels among patients with type II diabetes mellitus.

## Data Availability

The data that support the findings of this study are available on request from the corresponding author. The data are not publicly available due to information that could compromise the privacy of research participants.
